# Help seeking by male victims of domestic violence and abuse: an example of an integrated mixed methods synthesis of systematic review evidence defining methodological terms

**DOI:** 10.1186/s12913-020-05931-x

**Published:** 2020-11-26

**Authors:** Alyson L. Huntley, Eszter Szilassy, Lucy Potter, Alice Malpass, Emma Williamson, Gene Feder

**Affiliations:** 1 Centre for Academic Primary Care, Population Health Sciences, Bristol Medical School, University of Bristol, Canynge Hall, 39 Whatley Road, Bristol, BS8 2PS England; 2 The Centre for Gender and Violence Research, School for Policy Studies, Social Science Complex, 8 Priory Road, Bristol, BS8 1TZ England

**Keywords:** Mixed methods, Systematic review evidence, Domestic violence, Male victims, Help seeking

## Abstract

**Background:**

Domestic violence and abuse is a violation of human rights which damages the health and wellbeing of victims, their families and their friends. There has been less research on the experiences and support needs of male victims than those of women. Historically research on men’s experiences has not focused on what constitutes effective, needs-led service provision. The aim of this paper was to conduct an integrated mixed methods synthesis of systematic review evidence on the topic of help-seeking by male victims of domestic violence and abuse.

**Methods:**

An integrated mixed methods synthesis approach was taken to enhance our understanding of the complex phenomenon of help seeking by, and service provision to male victims. This process also identifies gaps in the evidence. Using previously identified systematic review data; mixed methods data from four primary-level service evaluation studies, along with expert and patient consultation were used to develop research propositions. Primary-level qualitative interview and survey data from 12 studies of men experiences were mapped onto the propositions to support them.

**Results:**

Fourteen propositions were composed. Seven propositions were supported or at least partly supported by the qualitative data. These supported propositions were used to make recommendations for policy and practice particularly concerning service preferences of male victims. The remaining seven propositions were not specifically supported by the qualitative data. These unsupported propositions were used to develop research recommendations concerning the need to further understand the potential blurred boundaries of victim–perpetrator, hybrid perpetrator-victim experiences, men who are/have been victims of childhood sexual abuse and determining the level of risk for men. They also highlight the need to produce better guidance for the response of the police & the criminal justice system. Finally, they highlight the need to produce the most appropriate service for men in terms of access, linkage, substance/alcohol abuse, mental health, sexuality, and race.

**Conclusion:**

Integrated mixed-methods synthesis of systematic review evidence is a relatively novel approach. This approach can lead to recommendations for policy and practice as well as highlighting gaps in the research agenda as shown in this example.

## Background

Domestic violence and abuse (DVA) is a violation of human rights which damages the health and wellbeing of victims, their families and friends. There has been less research on the experiences and support needs of men experiencing DVA than those of women survivors [1]. Historically much of the research on men’s experiences of DVA focused on prevalence and the forms of abuse suffered, and less on what constitutes effective, needs-led service provision [2]. More recent work has addressed these issues [3].

This integrated mixed methods synthesis (IMMS) builds on the systematic review evidence of four unpublished primary-level mixed methods service evaluation studies and a published synthesis of 12 primary-level qualitative interview and survey studies [4]. This IMMS enables us to further explore the barriers to help-seeking from formal support services in the broader context of men’s experiences.

There is a risk that systematic reviews steer the direction of research away from questions for which we do not have enough data, or which have not been asked. In this sometimes contentious area of research on male victims’ experiences of DVA, there are a number of important questions which need to be asked, but which can be obscured by what is already known. This paper seeks to broaden the scope of the conversation in this field by engaging both with what is known and what is not yet adequately known using a relatively novel mixed methods approach.

## Methods

### Scope

Help seeking by, and service provision to male victims of DVA. Definitions of relevant DVA terms can be found in Table 1.

**Table 1:** Definitions of terms used research concerning male victims of domestic violence and abuse

Term	Definition
*Domestic Violence and Abuse (DVA)*	Any incident or pattern of incidents of controlling coercive or threatening behaviour, violence or abuse between people aged 16 or over who are or have been intimate partners or family members, regardless of gender or sexuality [1].
*Referrers to DVA services*	Any professional person who can refer a victim of DVA to further formal support services even if it is an advocate who will then refer the person onto services. This can be a health care, social care, charity or criminal justice professional trained to perform this role.
*Signposting to services*	Victims may require different services outside what DVA services offer such as alcohol or drug services or specific mental health support. Although it is possible that some of these services may be combined with DVA service provision in some cases.
(*Blurred boundaries of) Victim/perpetrator*	Some perpetrators of DVA present to services as victims when that is not the case.
*Hybrid perpetrator-victims (experiences)*	Some victims may also be perpetrators and that this dual status might impact on the process of referral and the interventions these patients might require.

### Data source

We have previously conducted a systematic review of the relevant literature using standard methodology based on Cochrane principles [5]. This systematic review identified four mixed methods primary-level evaluations (quantitative and qualitative data) of the services for male victims of DVA and 12 primary-level qualitative studies of men’s experiences of help seeking and service provision. The protocol of this systematic review was published on the Prospero website [6]. The four primary-level service evaluations were not published in a systematic review due to the small number of studies. The 12 primary-level qualitative studies were synthesised and published as a standalone systematic review and qualitative synthesis paper [4].

### Methodological approach

Our IMMS was based on the mixed methods approach to systematic review evidence initially proposed by Dixon-Woods and colleagues and further developed by Heyvaert, Hanees and Obghena [7, 8]. Definitions of relevant IMMS methodological terms can be found in Table 2.

**Table 2:** Definitions relating to Integrated mixed methods synthesis (IMMS) of systematic review evidence

Term	Definition
***Primary level studies/evaluations***	Studies/evaluations in which researchers collect data (can be quantitative, qualitative or both) directly from their research participants.
***Systematic review***	A systematic review attempts to identify, appraise and synthesize all the empirical evidence [from primary level studies] that meets pre-specified eligibility criteria to answer a specific research question. Researchers conducting systematic reviews use explicit, systematic methods that are selected with a view aimed at minimizing bias, to produce more reliable findings to inform decision making [5].
***Integrated mixed methods synthesis of systematic review evidence (IMMS)***	Combining empirical evidence described in various kinds of primary level studies buy using various kinds of quantitative and qualitative synthesis techniques within a single systematic review to answer complex review questions and study complex topics and problems [8].
***Narrative Summary***	Narrative Summary is the selection, chronicling and ordering of evidence to produce an account of the evidence, and can integrate quantitative and qualitative evidence through narrative juxtaposition [7].
**Propositions**	Propositions are ideas or statements derived from the initial data which have not necessarily been subjected to empirical research *but are amenable to testing* through primary research.
***Propositions supported by (qualitative) evidence***	Propositions which can be supported at least in part by relevant evidence facilitate the IMMS approach and produce evidence that can be used for policy and practice.
***Propositions unsupported by evidence***	Propositions that cannot be supported by relevant evidence do not facilitate an IMMS and identify gaps in the evidence for research recommendations

In this synthesis we used a Narrative Summary approach facilitated by the development of propositions. Narrative Summary is often used in systematic reviews alongside systematic searching and appraisal techniques [7]. Narrative summary can vary from the simple description of findings through to more reflexive accounts that include commentary based on experience of the topic area. Narrative Summaries of the latter type can offering explanations that emphasise the sequential and contingent character of phenomena.

Despite methodological guidance, in practice Narrative Summary is still largely an informal approach and as such, is subject to criticism of its lack of transparency [7]. This is compounded by the fact that each Narrative Summary is different in nature based on the use of different combinations of evidence in a different format tackling a variety of evidence questions. As a consequence of this we also informed our methods from previously published IMMS of systematic review evidence [9–11].

In this IMMS, we composed propositions to explore service provision for male victims of DVA using the mixed methods primary-level evaluation study evidence identified in the systematic review and then looked to support these propositions with the evidence from the 12 primary-level qualitative interview and survey studies of men’s experiences.

### Methodology in practice

**Fig. 1:**
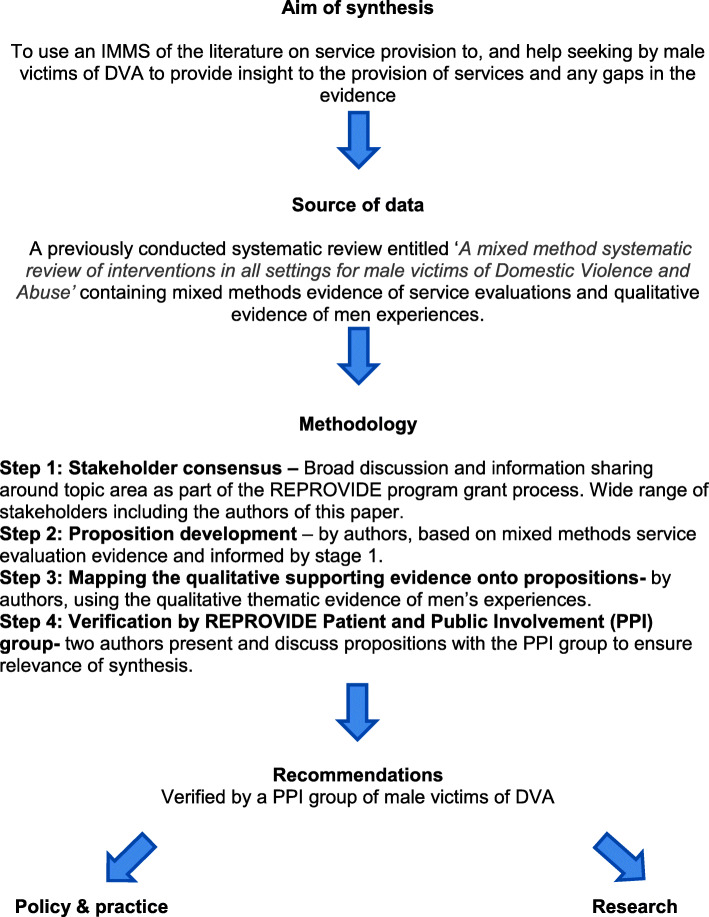
An integrated mixed methods synthesis (IMMS) of systematic review evidence for hep seeking for male victims of DVA

In practice, the above methodology was applied in individual and group work. (Fig. 1).

#### Step1) stakeholder consensus

The systematic review evidence was presented as part of a DVA stakeholder group (REPROVIDE Programme) consensus process for discussion. For the stakeholder group we identified experts in the field through existing contacts within the research team and through contacts of the REPROVIDE executive advisory group [12]. The group consisted of members from healthcare, safeguarding, police partnerships, academia, and third-sector organisations. Participants included three front line worker representatives of various independent charities specifically supporting male survivors, three front line health care professionals and two social care professionals working with survivors of DVA including male survivors.

The purpose of the consensus process was to explore and debate some controversial and difficult areas of practice. The meeting, which took place in September 2016, generated a constructive multi-professional debate around issues identified during the review process. The main outcome of the consensus process was to feed into the development of the General Practice-based training and advocacy support intervention for DVA as part of the REPROVIDE Programme [12]. However this process was also an invaluable information gathering and discussion activity for the present authors of this IMMS; assisting them in the formation of propositions in step 2.

#### Step 2) proposition development

The first author (AH) drafted some example propositions from the primary-level service evaluation study data and the process was discussed face to face with the rest of the authors using a previous published exemplar of IMMS to clarify the process [11]. Each author then used the data from the primary-level service evaluation studies to draft propositions individually in a Word document. Several of the authors have significant expertise in DVA research (GF, EW, ES). These propositions were collated by AH and discussed as a group to reduce redundancy. From this process one set of propositions was drafted by AH and recirculated. After individual consideration, the group met again to further refine the propositions.

#### Step 3) mapping the qualitative supporting evidence onto propositions

The primary-level qualitative interview and survey data and published synthesis was discussed by the authors as a group [4]. The qualitative themes and associated data were then initially mapped onto the propositions by AH. The propositions with supporting qualitative evidence in place were then discussed and edited as a group (face to face and by email) until all authors agreed with the IMMS achieved. Propositions not supported by qualitative evidence were also confirmed by the group.

#### Step 4) verification by REPROVIDE patient and public involvement group

These propositions were presented by the research team (GF, ES) to four members of the REPROVIDE male victim Patient and Public Involvement (PPI) group during a meeting in February 2017 to discuss their relevance and check that they reflected reality.

### Formulation of recommendations for, policy & practice and future research

Propositions with supporting qualitative evidence were used by the research team to formulate recommendations for policy and practice. Propositions without supporting qualitative evidence were used to discuss the gaps in the evidence for help seeking for male victims of DVA.

## Results

### Systematic review evidence

The previous *systematic review* ‘ A mixed method systematic review of interventions in all settings for male victims of Domestic Violence and Abuse’ identified four primary-level mixed methods evaluation studies [3, 13–15]. These primary-level evaluation studies described three interventions/service types. (Table 3) The systematic review also identified twelve relevant primary-level qualitative interview and survey studies of the experiences of male victims of *DVA* [16–27] (Table 4).

**Table 3:** Details of primary-level service evaluation studies

Study ID History of service Timeline	Details of services	Population during valuation	Objectives	Methods	Recommendations arising
**Robertson 2006** Dyn Final Evaluation Report. UK	Five guiding principles 1)That it is essential to develop essential services for GBT and heterosexual men which are effective in reducing risk and increasing safety 2) services must have a clear definition of domestic abuse 3)a clear screening protocol is essential in order to identify and respond appropriately to counter allegations 4) any service must have the capacity to risk assess referrals in order to identify those who are most at risk of experiencing abuse in the future 5) work with men who have experienced domestic abuse must take place within a multi-agency setting	Total of 171 men ‘The typical Dyn client is a white British male who is less than 40 years old, and reflecting the project’s status as a criminal justice agency has been referred to the project by the police. Where employment status is known, equal proportions are in full-time work as are unemployed.’	1) To document the types of services provided by the DYN project including an overview of the workload in the 12 mth period. 2) to understand the process of screening referrals to determine types 3) to identity the different levels of risk, fear, safety and relationships issues amongst the different types of men 4) To assess the impact of DYN on QoL & safety of men 5) to determine feedback men have about the DYN project 6) To identify links in working practice between DYN and WSU- benefits & challenges	• Case files • Case studies • Client Interviews • Key Informant Interviews • Participant observation	1)Maintaining existing provision so that all male victims have access to an appropriate service in Cardiff and further developing the capacity of the Dyn Wales / Dyn Cymru Helpline to ensure that male victims across Wales have access to support. 2) Conducting an empirical investigation into whether the FSU9 risk assessment form should be adapted specifically for male victims, 3) Conducting an empirical investigation into the respective risk profile of heterosexual men and their partners to inform service providers as to the nature of counter-allegations 4) Considering the development of a dedicated domestic abuse strategy for GBT men as their levels of risk and support uptake warrant different models of service provision. 5) Considering the development of a dedicated domestic abuse strategy for heterosexual men as their levels of risk and support uptake warrant different models of service provision. 6) Developing a set of agreed standards for work with men who have experienced domestic abuse to ensure that interventions identify and reduce risk while holding perpetrators to account.
**Debbonaire** **2008** [14] A report of an evaluation of the Men’s Advice Line UK	There is a helpline worker, supervised by the Respect Phone line Co-ordinator. The helpline is open six hours per day on three weekdays per week. Callers can contact the service by email. The helpline has Type Talk and Language Line capability enabling it to respond a wider diversity of callers.	All interviewees were male. Interviewees came from 15 different counties in England and Wales. 15 (68.2%) of the interviewees identified as “white”, “white British” or “white English”. 3 (13.6%) identified as Afro-Caribbean or Black, 2 (9.1%) identified as “Asian” and a further 2(9.1%) as “mixed race”. Age of caller (yrs) 21–29 4,.5% 30–39 54.5% 40–49 18.2% 50–59 22.7%	1. To evaluate the quality of the service provided to callers to the Men’s Advice Line (including male victims of domestic violence and professionals and other individuals wanting to help them) against the standards set in the model of work and any other relevant documents. 2. To evaluate the value of the service directly to callers and indirectly to other people such as their clients etc. 3. To provide a report detailing the findings of the above analysis and making recommendations if necessary about future development.	• Phone interviews • Email data gathering	That the Men’s Advice Line considers extending the opening hours for one night per week to 8 pm for a trial period to see if this helps callers who can’t ring during office hours.
**Debbonaire 2010** [15] Mens advice line client satisfaction report 2009–2010	Four staff and one coordinator now take calls. The opening hours of both lines are now 10 am – 5 pm with the lines closed between 1 and 2. Emails are also used as a method of providing advice, information and support on both lines.	The callers were from around England, Wales & Scotland. A total of 67 callers gave consent. Perpetrator 2 Victim 16 Perpetrator presenting as victim 9 Professional 4 Friend/family 4 Missing data 1 TOTAL (n) 36 **Ages** Under 18 0 0 18–21 0 0 21–30 1 3 31–40 6 3 41–50 14 1 51–60 7 1 Over 60 0 1 Missing 9 0 TOTAL 36 9 **Ethnicity** WhiteBritish 18 6 Bl’k/A-Carib 1 1 Indian/ Pakistani/ Asian 2 2 African 1 0 Other 3 0 Missing 11 0 Totals 36 9 **Sexuality** Heterosex 27 9 Lesbian/gay/bisexual 1 0 Missing 8 0	The survey focussed purely on satisfaction with the call. 1. To investigate the satisfaction of people contacting the Men’s Advice Line with the service they receive, by email or phone 2. To find out in particular if the callers/e-mailers were responded to promptly and courteously, whether or not they received help and advice they wanted, their overall levels of satisfaction, the type of advice and support they received and any suggestions for improving the service 3. To investigate this with a cross-selection of callers/mailers if possible 4. To prepare two short reports on the findings from each customer satisfaction survey, with, if appropriate, any recommendations for improving the service	• Phone interviews • Email data gathering	Nothing explicit
**Hester 2012** [3] Exploring the service and support needs of male, lesbian, gay, bi-sexual and transgendered and black and other minority ethnic victims of domestic and sexual violence	Of 111 service providers in the areas of London, the North West and South West of England who were possibly offering services to male, LGBT and/or BME victims of domestic or sexual violence, 76 responded. These represented a wide range of services across the voluntary, health and criminal justice sectors, as well as private solicitors and counsellors responded. This included 58 services from the voluntary, 15 from the statutory and three from the private sector.	The study included Male (heterosexual) victims of domestic and sexual violence Lesbian, gay, bi-sexual and transgender victims of domestic and sexual Violence Male black and minority ethnic victims of domestic and sexual violence and female BME victims of sexual violence **Service use samples** *Hetero/gay/bi/TG* Online 6,2,1,1 Interviews 5,2,0,0 Service data 22,0,0,0 Focus grp 0,0,015 BME 13,1,0,4 White 16,7,0,12 Other 4,0,0,0	The project aimed to begin to plug the existing knowledge gap via research with service providers and service users in three areas of England with the research focused on the extent and nature of both domestic and sexual violence, and the related service use and service needs, for under-researched population groups:	Victims of DVA • Face to face Interviews • Focus groups • On-line survey Service providers • . Face to face Interviews	The ‘Gold Book’ directory of domestic violence services should list services able to support heterosexual and/or BME male and LGBT victims of domestic violence. Service providers generally need training to understand and address domestic and sexual violence as these affect heterosexual and/or BME male and LGBT victims. There needs to be consideration of how support for heterosexual and/or BME male and LGBT victims might be located within existing services or through specialist provision. Third sector and specialist domestic and sexual violence services may be best placed to lead on this. Different forms of provision should also be considered, including helplines with long opening hours, and more outreach (mentioned by heterosexual men); web-based information There needs to be wider dissemination of risk assessment protocols for male victims of domestic violence in order to identify those who may also need support services for the perpetration of violence and abuse

**Table 4:** Details of primary-level qualitative interview and survey studies. First published in Huntley et al 2019. https://bmjopen.bmj.com/content/9/6/e021960.long

Author Date Country Setting/recruitment source	Study design	Research question/ aim of study	Participants demographics Age Ethnicity Education Marital status	Theoretical approach	Method of data analysis
**Bacchus** **2016** [16] **UK** Two generic sexual health clinics and one specialist sexual health clinic for (LGBT) patients in London.	Mixed method study survey & individual semi-structured interviews	To illustrate the use of a case series mixed methods for integrating interviews and survey data on gay and bisexual men’s experiences of negative & abusive behaviour in the context of intimate relationships.	*N* = 19 for interviews Mean age 39 years (range 21–57%) Ethnicity: Asian/asianbritish5.3% White 89.5% Other 5.3% paid employment 100%	Pragmatism (“what works as the truth regarding the research questions under investigation”)	The initial coding framework followed a deductive approach followed by open coding in an inductive process which allowed new themes to emerge
**Donovan** **2006** [17] **UK** Individuals were recruited from community groups and networks across the UK.	Mixed method study using UK wide survey, focus group and individual interviews	To provide a detailed picture of same sex domestic abuse, while at the same time being able to compare same sex and heterosexual experiences of such abuse	Five **focus groups** with lesbians, gay men and heterosexual women and men (*n* = 21). Semi-structured **interviews** with 67 individuals identifying as lesbian (19), gay male (19), heterosexual (14 women, 9 men), bisexual (3) or queer (3),.	None stated	No details
**Frierson 2014** [18] **PhD thesis** **USA** Participants identified via social service agencies and social organizations, as well as social media sites serving African American gay men	Qualitative interview study	To better understand how the intersections of race, gender, and sexual orientation inform African American gay males’ definition, experiences and help-seeking behaviours related to intimate partner violence.	13 male volunteers 18–40 yrs. identified as African American, Black, of African descent and/or biracial; identified their sexual orientation as gay or same-gender-loving; and had experienced at least one form of intimate abuse within a past and/or current relationship.	Constructivist grounded theory approach In addition, constructivist epistemological perspective as a part of the grounded theory approach was also used	Constant comparative analysis involves four phases of coding: initial coding, focused coding, axial coding, and theoretical coding.
**Hines 2010** [19] **USA** Men recruited via Domestic Abuse Helpline for Men and Women, a national IPV hotline specializing in men victims, Web sites, newsletters, blogs, and electronic mailing lists	Online questionnaire or telephone interview (same questions)	An in-depth, descriptive examination of men who sustained severe IPV from their women partners within the previous year and sought help.	299 men Mean age = 40.49 yrs. White 86.8% All in heterosexual relationships 56.5% currently in a relationship with their woman partners, 47.5% marriage followed by separation (17.9%). Relationships lasted on average 8.2 yrs.,	None stated	Qualitative responses were coded independently by 2 research assistants & any discrepancies were resolved by the first author**.**
**Hogan 2016** [20] **PhD thesis** **UK** Men recruited by domestic abuse services UK-wide (*n* = 2) mental health support services & drug/alcohol support services UK-wide (*n* = 9). Snowballing technique (*n* = 2) Presentation of preliminary findings at 2 UK conferences (*n* = 2) online support forums for male victims of domestic abuse & male victim support blogs (*n* = 8)	Qualitative interview study	To explore: (a) men’s experiences of female-perpetrated IPV, including their experiences of physical & psychological/emotional abuse; (b) men’s help-seeking experiences and/or their perceptions of utilising support services/support networks; and (c) barriers to men leaving their abusive relationship.	*n* = 23 Men > 18 yrs. who self-identified as a victim of female-perpetrated IPV. Race/ethnicity: White British (16) White other (5) British Pakistani (1) Black Afro Caribbean (1) Age: (range) 24–74 (mean: 47) Length of abusive relationship (range): 6 weeks – 31 years (mean: 12 years 5 months) Number of abusive relationships: One (17), Two (6)	Contextualist perspective (straddles essentialism & constructionism)	Thematic analysis was used to analyse the data following the six-phase process set out by Braun and Clarke (2006).
**Machado** **2017** **Portugal** Male victims of IPV in heterosexual relationships who had sought formal help from DV agencies	Participant’s demographics followed by semi-structured interview	To explore the experience of male Portuguese victims who had sought help for their victimization.	*N* = 10 mean age 51.6 yrs. (range 35-75 yrs) 50% had < 12 yrs. education *n* = 6 employed *n* = 4 retired *n* = 3 lower class *n* = 3 lower middle class *n* = 2 middle class *n* = 2 upper middle class	None stated	Thematic analysis. Transcripts analysed based on emerging themes. To ensure validity and credibility of results, different strategies were adopted, including constant comparative analysis & a dense description of the meanings.
**McCarrick** **2016** [22] **UK** Charitable agency that support male victims & via advertisements placed on a website.	Unstructured Face-to-face and Skype qualitative interviews.	To explore men’s experience of the UK Criminal Justice System (CJS) following female-perpetrated IPV	6 male participants (45-60 yrs) over 18 yrs. and having experienced female-perpetrated intimate partner violence (IPV) and subsequent involvement with the CJS	Interpretative phenomenological analysis	Interviews were transcribed and analysed by the researcher in a process of reflexivity.
**Morgan** **2014** [23] **UK** Men recruited from GP surgeries in south west of England	Cross-sectional survey & follow-up interviews investigating the impact of men’s relationships on their health	To expand the current body of knowledge on male help-seeking in relation to DVA by measuring & characterising help-seeking practices.	No demographic details	Grounded theory approach	A coding framework was used that was developed in conjunction with colleagues across the wider study.
**Morgan** **2016** [24] **UK** Participants recruited from websites of UK-based organisations supporting male victims of IPV.	Semi-structured interview methodology	To investigate male victims’ experiences of female-perpetrated IPV	*n* = 7 Researchers asked participants not to disclose their demographics of age, occupation etc. range of length of relationship 3-13 yrs. range of time since relationship finished 18mths-14 yrs	interpretative phenomenological (theory) analysis (IPA)	The scripts were transcribed verbatim from audio recordings using the Jefferson technique & analysed using IPA.
**Simmons** **2016** [25] **Sweden** Primary health care	Qualitative interview study	To develop a theoretical model concerning male victims’ processes of disclosing experiences of victimisation to health care professionals in Sweden.	Informants were recruited from respondents in a quantitative study of being subjected to IPV, Ill health, seeking behaviour conducted in men and women in the general population (*n* = 1510, response rate 37%) and at two primary health care centres (*n* = 129, response rate 70%) recruited from the random population sample.	Constructivist grounded theory	After each interview, codes & categories created in analysis helped to choose the next informant, and the guide was modified to explore related topics & elaborate categories. A constant comparative analysis both within an interview and between interviews. Next focused coding was used in which most significant line-by-line codes were used
**Tsui** **2010** [26] **USA** 960 DVA services across USA	Survey consists of five closed ended questions two open-ended questions & 13 demographic questions	To examine the needs of male victims to identify factors that block men from seeking help.’	Sixty-eight agency representatives responded. Mean age 43 yrs. 72% female 81% Caucasian 7.3% Hispanic 5.9% African American 88.2% held an academic degree 84% were professional or managerial staff in the DVA organizations	None stated	Qualitative data were coded to thematic units. Similar units with meaning related to male victims were assigned to categories and organized into themes and further reviewed by research team to enhance face & content validity.
**Valentine** **2013** [27] **USA** Men recruited from university-affiliated, outpatient HIV/AIDS primary care clinic	Qualitative interview study	To qualitatively explore the ways in which such men find meaning following their experiences of partner abuse	(*n* = 28) ≥18 yrs., English-speaking, currently receiving HIV-related care at the clinic site. Mean age 43.6, *SD* 5 6.2 Male 24, Transgender 4. Gay 23, Bisexual 3 Bi spirit 1, Other 1 Currently in relationship 12 Living with partner 6 Relationship status unknown 10 White/European 13 Black/African American 8, Latino/Hispanic 2 American, Indian/Alaskan Native 3 Biracial/multiracial	None stated	Data analysis was conducted by a team who contributed to the reading, coding, categorizing, Consistent with conventional content analysis, no codes, categories, or themes were specified a priori (Hsieh & Shannon, 2005). To establish dependability (Morrow, 2005), all three reviewers met to compare codes and reach a consensus.

#### Service evaluation studies

Three papers describe two standalone male victim support services [13–15]. The fourth paper was a broader evaluation of service providers in selected areas of England [3].

- 1.The Dyn project located within the Women’s safety unit Cardiff, UK began accepting referrals in January 2005, although relocated to a different site subsequently [13]. The evaluation was for a 12-month period and included data from 171 men. Quantitative and qualitative data was collected via case files, case studies, client interviews, key informant interviews and participant observation. This evaluation made six main recommendations for practice.
- 2 & 3. There are two separate evaluation reports of a men’s advice line based in London [14, 15]. The first was conducted in February 2007 following a relaunch of an advice line for male victims of DVA by the organisation RESPECT [14]. Interviewees were recruited from mid-January-mid April 2008. All 21 interviewees were male and came from 15 different counties in England and Wales. Quantitative and qualitative data collection was via phone interviews and email. These data were narratively presented, and the key recommendation was that the service considered extending the opening hours for one night per week to 8 pm for a trial period to see if this helped callers who could not ring during office hours.

The second report was in 2010 and was a satisfaction report conducted November to December 2009 and late January- early February 2010 [15]. Since the first evaluation the helplines had expanded in staff numbers and opening hours. A total of 67 callers gave consent and were from England, Wales & Scotland. Quantitative and qualitative data were collected by via phone interviews and email. There were no specific recommendations from this report.

- 4. The fourth publication described a pan service research evaluation in the areas of London, the North West and South West of England exploring the service and support needs of male, lesbian, gay, bi-sexual, transgendered, black and other minority ethnic victims of domestic and sexual violence [3]. The evaluation included male (heterosexual) victims of domestic and sexual violence. Quantitative and qualitative data collection was via face to face interviews, focus groups and on-line surveys with victims of DVA as well as face to face interviews with service providers. This evaluation made six main recommendations for practice.

#### Qualitative interview and survey studies

The searches identified 12 relevant primary-level qualitative studies of the experiences of male victims of DVA. Six studies were conducted in the UK, four in the USA and one each in Sweden and Portugal and all were published between 2006 and 2017.

The synthesis of the qualitative interview and survey data is published [4]. In this synthesis, we generated key themes in two main categories (1) barriers to help-seeking and (2) experiences of interventions and support. We derived five themes on the barriers to initial disclosure and help-seeking by male victims of DVA. Three themes were closely related: fear of disclosure, challenge to masculinity and commitment to relationship. Other themes were diminished confidence/despondency and the invisibility/perception of services.

Four themes emerged relating to experiences of interventions and support: initial contact, confidentiality, appropriate professional approaches and inappropriate professional approaches. The theme of confidentiality is closely linked to the themes of appropriateness of professional approaches. (Additional file 1).

##### Propositions and supporting evidence

The development process described in the methods produced 14 propositions. (Table 5) Seven propositions were fully or at least partly supported by the qualitative data and an IMMS was achieved. The remaining seven propositions were not supported specifically by the qualitative data and could not undergo an IMMS.

**Table 5:** Contribution of the papers to the responses to the propositions

Propositions	Papers	Most relevant themes
1. *It is important to understand who are acceptable referrers to a male victim service*	Bacchus 2016 [16] Donovan 2006 [17] Frierson 2014 [18] Hogan 2016 [20] Morgan 2014 [23] Simmons 2017 McCarrick 2016 [22]	Initial contact Appropriate professional approach Inappropriate professional approach
2. *If men’s services are linked to women’s services, we need to know which features of the ‘shop front’ are important so as not to put men off seeking help*	Donovan 2006 [17] Frierson 2014 [18] McCarrick 2016 [22] Tsui 2010 [26]	invisibility/perception of services
3. *There is a need to publicise our services for male victims to appeal to men of all backgrounds.*	Donovan 2006 [17] Frierson 2014 [18] Hines 2010 [19]	invisibility/perception of services
4. *It is important that we understand what male victims mean by “practical help” or “advice about what to do”*	Hines 2010 [19] Simmons 2017 Machado 2017 Morgan 2014 [23]	Fear of disclosure Commitment to relationships
5. *A sensible approach to initially provide practical advice to male victims which then may help them to talk about their emotional issues.*	Bacchus 2016 [16] Frierson 2014 [18] Morgan 2014 [23] Tsui 2010 [26] Simmons 2017	Appropriate professional approaches Diminished confidence and despondency
6. *Understanding what male victims tell us about the ways in which they like to seek help is important.* E.g. *online, by phone, continuity of contact*	Bacchus 2016 [16] Frierson 2014 [18] Hogan 2016 [20] Morgan 2014 [23] Simmons 2017	Confidentiality Appropriate professional approaches
7. *There is a need to know what the core training needs of service providers, and ongoing support needs of male survivors are.*	Bacchus 2016 [16] Donovan 2006 [17] Frierson 2014 [18] Hogan 2016 [20] McCarrick 2016 [22] Morgan 2014 [23] Morgan 2016 [24] Simmons 2017 Tsui 2010 [26]	Confidentiality Appropriate professional approaches Inappropriate professional approaches
8. *It is important that we use the most appropriate approach to the potential blurred boundaries of victim –perpetrator (to provide support and not to make the man feel like it is surveillance)*	No significant contribution from papers	No relevant themes
9. *There is a need to understand the appropriate approach to hybrid perpetrator-victim experiences.*	None	No relevant themes
10. *There is a need to understand the appropriate approach to discussing the current experiences of men who are/have been victims of childhood sexual abuse.*	None	No relevant themes
11. *There is a need to understand the most appropriate and adequate way of determining the level of risk a man is at (bearing in mind current risk tools are underdeveloped)*	None	No relevant themes
12. *There is a need to know if it is possible to have a single service (point of access) to provide appropriate support and linkage to other services to male victims from all backgrounds.*	None	No relevant themes
13. *For men with experience of substance/alcohol abuse or mental health problems we need to know how it is best to signpost to relevant services.*	No significant contribution from papers	No relevant themes
14. *There is a need to understand how linkage/co-ordination between services supporting male victims can be maximised.*	No significant contribution from papers	No relevant themes

#### Propositions 1-7

These propositions were fully or at least partly supported by the qualitative evidence and an IMMS was achieved leading to policy and practice recommendations.

**Proposition 1:** It is important to understand who are acceptable referrers to a male victim service.

### Relevant qualitative themes

This proposition most closely relates to the qualitative themes of *Initial contact*, *Appropriate professional approach* and *Inappropriate professional approach* and is specifically supported by qualitative data from seven primary qualitative studies covering community based DVA services as well as primary health care and sexual health services [16–18, 20, 22, 23, 25].

### Supporting evidence

The qualitative findings suggest a mixed response by men to referral from primary health care, with some men not perceiving it as a source of help as well as doubting the compassion and approach to confidentiality of general practitioners [16, 25]. Other men felt that general practice is a good place to disclose DVA and be referred, and that this process is facilitated by continuity of care and an individual approach by clinicans [16, 23, 25].

The data around the response of police were also mixed, but generally the criminal justice system is not perceived as receptive to disclosure of DVA and referral with the fear of being wrongly accused of perpetration and inappropriate reaction in some cases [17, 18, 20, 22]. There are no qualitative data on the acceptability of referrals from other professional support services in the community, such as drug and alcohol workers.

There was only one study which recruited men from sexual health clinics and these men appeared to have no problems with non-medically trained advisors supporting and referring them on [16].

There is a consistent theme that female professional help in referral is acceptable, if not preferable for male victims of DVA with no comparable discussion around male professional help in these settings [16, 20, 23, 25].

### P1 summary

There is no clear message about preference on disclosing to professionals in health care settings and being referred, and mixed feelings concerning the police. There is a lack of evidence on community support services. However, there is a consistent message that female professional help is acceptable.

**Proposition 2:** If men’s services are linked to women’s services, we need to know which features of the ‘shop front’ are important so as not to put men off seeking help.

### Relevant qualitative themes

This proposition is most related to the qualitative themes of *Invisibility/perception of services* and is specifically supported by qualitative data from four of the primary-level qualitative studies [16, 18, 22, 26].

### Supporting evidence

There was no significant discussion in the studies around the need for separate services for male victims, although it was specifically mentioned in the Tsui study [26]. What appeared to be more important to men was the lack of visibility and targeting of services towards men, and thus a perception, or perhaps the reality of a lack of access to such services [16, 22, 26]. This also links to the discussion by men that there is a lack of public awareness and political engagement of male victims of DVA [26].

### P2 summary

There is no direct evidence about what a ‘shop front’ of services for male victims should look like; rather the evidence is that men are more concerned about access to services in the first instance.

**Proposition 3:** There is a need to publicise our services for male victims to appeal to men of all backgrounds.

### Relevant qualitative themes

This proposition broadly relates to the qualitative theme of *Invisibility/perception of services* and is specifically supported by qualitative data from three of the primary-level qualitative studies [17–19].

### Supporting evidence

Donovan and Hester summed up the issue discussed in several studies as ‘heterosexism of individual professionals’ which can lead to a sense of isolation and a low expectation of services from the LGBT population [17]. There is some mention of ethnic barriers [19] to help seeking but the majority of the men in the studies were of White background with the exception of the Frierson study [18].

### P3 summary

There is a lack of evidence of services being publicised to appeal to men of all backgrounds. Issues are raised about bias in favour of white, heterosexual males.

**Proposition 4:** It is important that we understand what male victims mean by “practical help” or “advice about what to do.”

### Relevant qualitative themes

This proposition relates in part to the qualitative themes of *Fear of disclosure* and *Commitment to relationships* and is specifically supported by four of the 12 primary qualitative studies [19, 21, 23, 25].

### Supporting evidence

Some of the common fears of men disclosing DVA were losing the custody of their children, financial implications, and possibility of having ‘nowhere to go’. This suggests that legal and housing advice would be welcome [19, 25]. Another common expression of help was around the desire to help their partner. In other words, potentially getting their partners help for their behaviour [19]. The clinicians and DVA support services would not have to necessarily support both parties on their own but potentially facilitate access/ refer to appropriate support for the partner.

In Machado and colleagues’ primary-level interview study male heterosexual victims of DVA report how informal help-seeking leads on to formal help-seeking but they also say *‘*However, the overwhelming majority of participants rated formal sources as unhelpful, especially the services of the judicial system’. [21] Conversely, men reported that they had received valuable support from friends, family and colleagues at work. However not all men’s accounts of accessing informal help were not always positive [23].

### P4 summary

The evidence gives a strong steer as to what is important to men: children, money, housing, and their partners situation featuring in their narratives. The evidence also suggests they may not seek formal support for these issues but rather look for help from family and friends.

**Proposition 5:** A sensible approach to initially provide practical advice to male victims which then may help them to talk about their emotional issues.

### Relevant qualitative themes

This proposition most closely relates to the qualitative themes of *Appropriate professional approaches* and *Diminished confidence and despondency* and is specifically addressed by five of the original 12 primary-level qualitative studies [16, 18, 23, 25, 26].

### Supporting evidence

This proposition would appear to be sensible as we know that often a crisis has to occur before men seek help. Therefore emergency legal, financial and medical help would be the most practical initial response [18, 25]. The qualitative studies also report that men can minimise or downplay their experiences, so providing practical advice is likely to be less daunting than a specific therapeutic intervention [16, 18, 26]. The primary-level qualitative studies also report that professional interaction and continuity of contact (as in general practice), gives time for men to feel they can trust the professional and be comfortable in discussing their situation in more detail [16, 23].

### P5 summary

Whilst there is no direct evidence that providing practical advice first facilitates emotional support, some indirect evidence regarding men’s interaction with services suggests this is a sensible strategy.

**Proposition 6:** Understanding what male victims tell us about the ways in which they like to seek help is important. E.g. online, by phone, continuity of contact

### Relevant qualitative themes

This proposition most closely relates to the qualitative themes of *Confidentiality* and *Appropriate professional approaches* and is specifically supported by five of the twelve primary-level qualitative studies [16, 18, 20, 23, 25].

### Supporting evidence

No specific routes of help were discussed by the men in the included studies but there was a strong theme of the need for confidentiality and physical privacy in disclosing [16, 20, 23, 25]. Men seemed to appreciate continuity of care from services, and to prefer disclosing to female professionals [16, 18, 23].

### P6 summary

There is no evidence in the primary-level qualitative studies as to the preference of ways e.g. phone, face to face men would like to communicate. However, the need for both confidentiality and physical privacy featured strongly.

**Proposition 7:** There is a need to know what the core training needs of service providers, and ongoing support needs of male survivors are.

### Relevant qualitative themes

This proposition most closely related to the qualitative themes of *Confidentiality*, *Appropriate professional approaches* and *Inappropriate professional approaches* and is specifically supported by nine of the 12 primary-level qualitative studies [16–18, 20, 22–26].

### Supporting evidence

These themes from the qualitative synthesis can inform guidance on the approach to service provision via 1)the importance of a private, confidential space to talk in; 2) the importance of being believed and listened to; 3) that the professional is knowledgeable about DVA (for example, because someone has left their partner does not mean they are not at risk); 4) not to avoid difficult conversations; to take disclosure seriously and not to use humour; 5) professionals should acknowledge sexuality and ethnicity; 6) to avoid assumptions about sexuality (particularly heteronormative assumptions) and 7) appropriate transparent signposting. The qualitative themes demonstrate the importance of a facilitative environment, sensible timing and effective communication skills in trust building and effective immediate response [4].

### P7 summary

The evidence gives a strong steer as to what men expect out of a professional service both in terms of practical requirements e.g. confidential space and emotional support.

#### Propositions 8-14

These propositions were not supported by the primary-level qualitative study evidence and an IMMS was not achieved, identifying gaps in the evidence. Some of the proposition topics were briefly mentioned in the qualitative papers but with no evidence available to use for an IMMS*.*

**Proposition 8:** It is important that we use the most appropriate approach to the potential blurred boundaries of victim –perpetrator (to provide support and not to make the man feel like it is surveillance)

McCarrick and colleagues interviewed men who were frustrated and distressed by the confusion over victim/perpetrator role by the criminal justice system but no practical recommendations on this issue were discussed [22].

**Proposition 9:** There is a need to understand the appropriate approach to hybrid perpetrator-victim experiences.

**Proposition 10:** There is a need to understand the appropriate approach to discussing the current experiences of men who are/have been victims of childhood sexual abuse.

**Proposition 11**: There is a need to understand the most appropriate and adequate way of determining the level of risk a man is at (bearing in mind current risk tools are underdeveloped).

**Proposition 12:** There is a need to know if it is possible to have a single service (point of access) to provide appropriate support and linkage to other services to male victims from all backgrounds.

**Proposition 13:** For men with experience of substance/alcohol abuse or mental health problems we need to know how it is best to signpost to relevant services.

McCarrick mentions that mental health issues and lack of confidence e.g. post-traumatic stress disorder may prevent men accessing services [22].

**Proposition 14:** There is a need to understand how linkage/co-ordination between services supporting male victims can be maximised*.*

There were no significant primary-level qualitative data to provide any specific support to this proposition although it was acknowledged as an important issue in a couple of the studies [26, 27]. Two further studies included men who talked about the importance of peer support and wanting to give something back [20, 22]. This could be a mechanism for linking men together and sharing knowledge of services. It was pointed out by one man that it was important that the service signposted to was useful or there was no point in referring on [20].

## Recommendations for policy & practice and future research

**Fig. 2:**
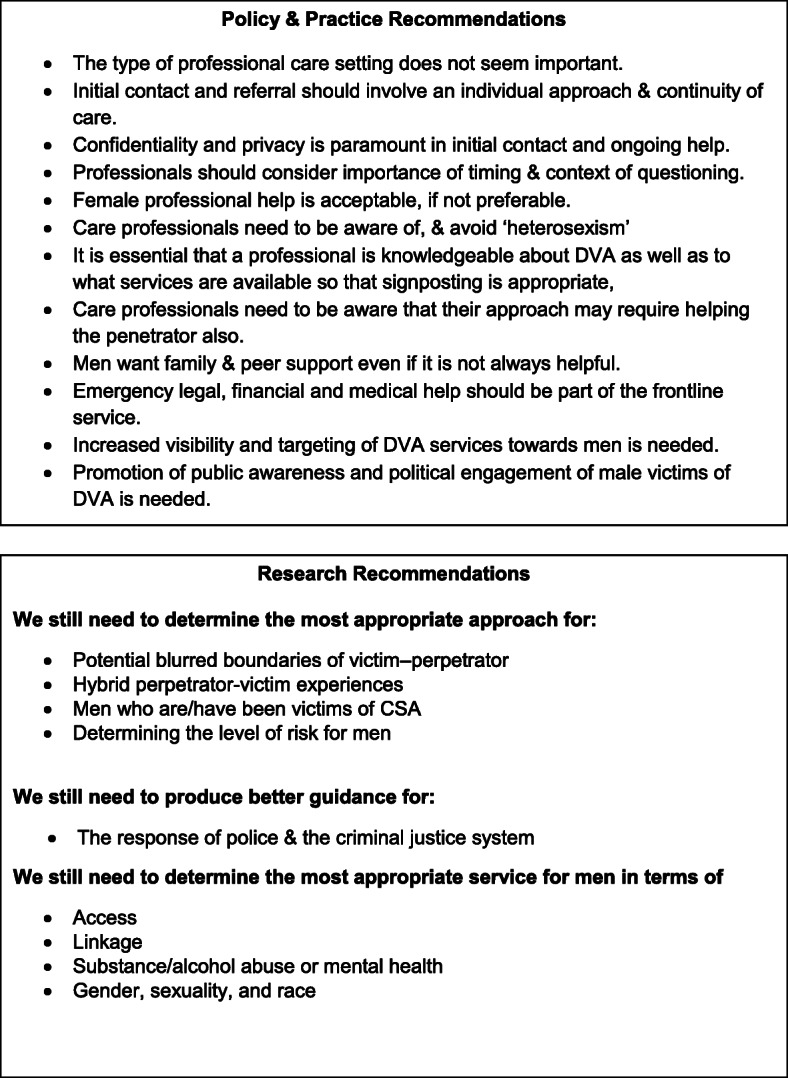
Recommendations for policy & practice and future research

Whilst the evidence and evidence gaps we have articulated are likely to be universally relevant to male victims of DVA across the world, country-specific policy, practice and service provision does differ. As all four of the primary-level evaluation studies were based in the UK, our policy and practice recommendations are also UK orientated. That said, the fundamentals of our recommendations are likely to be broadly applicable to many countries. Recommendations were derived from the propositions by the one author (AH) and discussed and modified by the other authors until consensus was reached**.** (Fig. 2).

## Discussion

This is the first IMMS of systematic review evidence on help seeking by and service provision to male victims of DVA. We have generated recommendations from our synthesis based on what men would ideally want from a service and high-light significant gaps in the evidence for service provision. Our work adds to the development of IMMS within health and social care research by promoting the use of this relatively novel methodology, defining terms and extending the methodology by using extensive stakeholder involvement.

In this discussion, we firstly debate the service provision in light of the evidence of the needs and actual experience of service provision by male victims of DVA and secondly contextualise the gaps in the evidence identified and make recommendations on the future of research in these areas. We acknowledge that these two tasks are not mutually exclusive.

### Policy and practice recommendations based men’s needs and actual experience

Propositions 1-7 have informed policy and practice recommendations regarding initial contact and approach, referral and ongoing care of male victims of DVA by professionals. They also identify the wider issues of awareness of male victims of DVA both by professional services and the public alike. This indicates a role for primary prevention efforts and wider media awareness campaigns around men’s health.

Society is becoming more aware of male victims of DVA, and research, practice and policy are progressing, However, there are still many obstacles, not in the least unrelenting financial pressure on the DVA service sector and now in 2020 we have the impact of the Covid-19 crisis [28].

We need to promote an intersectional approach in existing front-line organisations as well as supporting the development of new male victim needs-led support schemes. Robust evidence of effectiveness of interventions and appropriate resources supporting the running of these organisations/schemes are critical.

### Evidence gaps and research recommendations

Seven of our 14 propositions were not addressed by our IMMS (P8—14) and thus are the basis of the research recommendations. In part, it is important to acknowledge the issues are extremely difficult to address. However, equally we are aware of practice and to a lesser extent research in progress with many of these issues.

*Propositions* 7-10 deal with how professionals approach the challenging issue of getting an accurate picture of a man’s relationship and experience of DVA. Being aware of any childhood or previous abuse history and the initial contact of male victims with services.

Whilst there is a lack of formal research internationally, services have been developed in the USA, Canada, some Scandinavian countries and the Antipodes. Respect, London UK (http://respect.uk.net/) have developed a toolkit and national standards for working with male victims of DVA [29, 30]. This toolkit covers supporting men of all sexualities as well as guidance on ‘identifying who is doing what to whom and with what effect’ and includes a checklist. This toolkit is based on the primary-level evaluation and participant satisfaction studies of the Respect telephone line [14, 15].

Male victims of DVA are disturbed (and avoid services) because of their perception that they can be treated as perpetrators, yet we know from both the female and male victim data that their perpetrators sometimes present as victims at various points [3, 31]. Whilst it is important to determine how to address the needs of these perpetrators who sometimes present as victims, these approaches are likely to be rejected by them. True male victims of DVA are equally likely to find these approaches offensive. The DRIVE project currently in progress aims to impact the lives of victims, children and perpetrators by offering a multi-agency intervention and ensuring that the criminal justice system provides a robust response and is likely a potential future source of evidence for the evidence gaps identified by this analysis [32].

*Propositions* 11-14 ask about service provision for male victims of DVA from all backgrounds whether it is possible to have a single service/point of access, whether these services are linked, and proposition 12 specifically tackles substance/alcohol abuse and mental health support.

In the UK, helplines set up for male victims of DVA are likely to cover wide geographical areas but refer to local services. Blanket local provision would be difficult to justify financially as it is known that relatively small numbers of men are known to access these services [33].

In terms of the high prevalence of alcohol and drug problems in DVA situations, work in the UK focuses on female victims and ongoing work seeks to develop an integrated substance abuse/DVA approach to tackling these challenging issues [34, 35].

Linkage and communication between DVA and related services are difficult as the needs of male victims are diverse, and different services have different approaches. However, as our research shows, and the activities of DVA organisations are evolving, awareness surrounding intersectionality and the multiple barriers to help seeking and disclosure by male victims of DVA is increasing. This in turn can contribute to not only to a more nuanced understanding of the experiences of male victims and the obstacles to support but can also lead to a more joined up approach.

The findings of this *IMMS* fed into the development of the IRIS+ integrated general practice-based training and advocacy support intervention program [33]. IRIS + is designed to engage general practices in addressing the needs of women and men experiencing or perpetrating DVA and their children, offering affected patients a direct referral pathway to specialist services [36]. IRIS+ includes elements of training on and specific support for male victims. The feasibility, acceptability and value for money of IRIS+ is currently being tested and the findings of this study will be an important addition to the evidence base of joint primary health care and specialist DVA sector response to male victims.

## Strengths and limitations

This IMMS is based on data obtained from a robust systematic review and has been conducted by an experienced team both in terms of the evidence synthesis methodology and the topic area of DVA. This synthesis is also a component part of the REPROVIDE programme and gains from the expertise of that group [12]. The IMMS approach is becoming established within the evidence synthesis community as a useful tool for understanding complex situations as well as identifying any gaps in the evidence.

The limitation of such an approach are that proposition development is a subjective process reliant on the expertise and objectivity of the proposers. This can be counteracted at least in part by transparency of process and involving a wide variety of stakeholders, as well as consulting with a PPI group. Ideally, we would have consulted the male victim PPI group early in the process during the development of the propositions as opposed asking for their confirmation and comments on them. We were however restricted by the timings and availability of these meetings. In future analysis, we would endeavour to involve all parties in the initial development of the propositions.

Production of an IMMS, is relatively lengthy, requiring conduct of the systematic review and initial synthesis of at least part of the data before moving on to the more complex mixed methods analysis. Careful thought is needed to ensure that a mixed methods synthesis is justified within a project. Our synthesis was part of a large programme of work and we feel justified that its output has not only fed into intervention development but has also high-lighted important evidence gaps in the area of male victims of DVA that need to be addressed in future research. We are aware because of the length of this process there is new evidence which could be assimilated into this IMMS. Three multi-country studies by the same research team have been published in 2020 [37–39].. One of these new studies reenforces the findings of our IMMS on help seeking by male victims and service provision [37]. The second study specifically examines male victims experiences of the criminal justice system and this would add new material to our IMMS [38]. The final study focuses on male victims experiences of female perpetrated violence [39]. In an ideal world we would update this IMMS but we are confident that the evidence summary and conclusions still stands in 2020.

## Conclusions

Mixed methods synthesis of systematically reviewed evidence is an appropriate and useful research tool to further knowledge. Application of this approach to help- seeking by and service provision to male victims of DVA has informed recommendations for policy and practice as well as highlighting gaps in the research agenda.

## Supplementary Information

**Additional file 1 Appendix 1.** Themes and subthemes from qualitative synthesis.

## Data Availability

Full data extraction and synthesis data are available from the authors although the source data is already in the public domain.
