# Supplementary material for: Help seeking by male victims of domestic violence and abuse: an example of an integrated mixed methods synthesis of systematic review evidence defining methodological terms
Source: BMC Health Serv Res. 2020 Nov 26;20:1085. doi: 10.1186/s12913-020-05931-x (PMC7689389; doi:10.1186/s12913-020-05931-x)
Supplement: Supplementary file 1 — Additional file 1 Appendix 1. Themes and subthemes from qualitative synthesis. [file 12913_2020_5931_MOESM1_ESM.docx]

**Appendix 1:**

Themes and subthemes from qualitative synthesis

First published in Huntley *et al* 2019 https://bmjopen.bmj.com/content/9/6/e021960.long

| **Phase 1:** **Barriers to help-seeking** | **Studies providing data** |
| --- | --- |
| **Themes** |  |
| Fear of disclosure (internal & external pressures) | Frierson 2014  Hines 2014  Hogan 2016  Machado 2017  McCarrick 2016  Morgan 2014  Morgan 2017  Simmons 2017  Tsui 2010  Valentine 2013 |
| Challenge to masculinity | Frierson 2014  Hines 2014  Hogan 2016  McCarrick 2016  Morgan 2016  Simmons 2017  Tsui 2010 |
| Commitment to relationships | Hines 2014  Simmons 2017 |
| Diminished confidence and despondency | McCarrick 2016  Tsui 2010  Valentine 2013 |
| Invisibility/perception of services | Bacchus 2016  Frierson 2014  McCarrick 2016 |
| **Phase two: Experiences of interventions and support** |  |
| **Themes** |  |
| Initial contact (tipping the balance) | Donovan 2006  McCarrick 2016  Morgan 2014  Simmons 2017 |
| Confidentiality | Bacchus 2016  Frierson 2014  Hogan 2016  Morgan 2014  Simmons 2017 |
| Appropriate professional approaches | Bacchus 2016  Hogan 2016  Machado 2017  Morgan 2014  Simmons 2017 |
| Inappropriate professional approaches | Donovan 2006  Frierson 2014  Hogan 2016  McCarrick 2016  Machado 2107  Morgan 2016 |
